# Gastrointestinal Infection Could Be New Focus for Coronavirus Diagnosis

**DOI:** 10.7759/cureus.7422

**Published:** 2020-03-26

**Authors:** Massimiliano Cipriano, Enzo Ruberti, Andrea Giacalone

**Affiliations:** 1 Department of Laparoscopic Surgery, Umberto I General Hospital, Medical School Sapienza University, Rome, ITA; 2 Neurology, Sapienza University, Rome, ITA; 3 Industrial Engineering Technologies for Sports Medicine and Rehabilitation, University of Rome Tor Vergata, Rome, ITA

**Keywords:** covid-19, 2019 novel coronavirus, sars-cov-2, gastrointestinal infection, enteric symptoms, acute respiratory syndrome, ace2, diarrhea

## Abstract

It's not news to tell you that the coronavirus, known as COVID-19, is a worldwide pandemic. The initial outbreak of this novel virus in Wuhan in the Hubei province of China, first described in December 2019, has since moved on to being declared a pandemic by the World Health Organization.

The classic description of COVID-19 is a respiratory illness that manifests with fever, dry cough, and dyspnea on exertion. However, gastrointestinal (GI) complication of COVID-19 is emerging as well. This was observed with similar viral respiratory illnesses, such as severe acute respiratory syndrome (SARS), which emerged in 2003, and the Middle East respiratory syndrome (MERS), which emerged in 2012.

In a recently published, single-center case series of 138 consecutive hospitalized patients with confirmed COVID-19, investigators reported that approximately 10% of patients initially presented with GI symptoms, prior to the subsequent development of respiratory symptoms. Common and often very subtle symptoms included diarrhea, nausea, and abdominal pain, with a less common symptom being nonspecific GI illness. New studies are expanding our understanding of the possible fecal transmission of COVID-19. Assessment by polymerase chain reaction (PCR) has provided evidence of the virus in the stool and the oropharynx outside the nasopharynx and respiratory tract. Virus in the stool may be evident on presentation and last throughout the course of illness resolution for up to 12 days after the respiratory virus evidence is gone. In fact, in one of the most recent studies looking at 73 patients, approximately 24% remained positive in their stool for evidence of the virus, though not necessarily infection, after showing negative in respiratory samples.

The Centers for Disease Control and Prevention (CDC) recommends that after two negative respiratory tests separated by ≥ 24 hours, patients can be dismissed from having transmissibility infection risk for COVID-19.

The potential for fecal-oral transmission of COVID-19 needs to be strongly considered. Considering these cases and the lessons from SARS, many authors recommend that real-time reverse transcriptase-polymerase chain reaction (rRT-PCR) testing for severe acute respiratory syndrome coronavirus 2 (SARS-CoV-2) from feces should be performed routinely in SARS-CoV-2 patients.

## Introduction and background

As of March 11, 2020, 118,598 cases of COVID-19 were reported worldwide by more than 100 countries. Since late February, the majority of cases reported are from outside China, with an increasing majority of these reported from European Union (EU)/European Economic Area (EEA) countries and the UK. The Director-General of the World Health Organization declared COVID-19 a global pandemic on March 11, 2020. All EU/EEA countries and the UK are affected, reporting a total of 17,413 cases as of March 11. Seven-hundred eleven cases reported by EU/EEA countries and the UK have died. Italy represents 58% of the cases and 88% of the fatalities. The current pace of the increase in cases in the EU/EEA and the UK mirrors trends seen in China in January to early February and trends seen in Italy in mid-February [1]. The end of 2019 was marked by the emergence of a novel coronavirus, severe acute respiratory syndrome coronavirus 2 (SARS-CoV-2), which caused an outbreak of viral pneumonia (COVID-19) in Wuhan, China [2]. Coronaviruses are a family of single-stranded enveloped RNA viruses that are divided into four major genera. The genome sequence of SARS-CoV-2 is 82% similar to severe acute respiratory syndrome coronavirus (SARS-CoV) and both belong to the β-genus of the coronavirus family [3-4].

Human coronaviruses, such as SARS-CoV and Middle East respiratory syndrome coronavirus (MERS-CoV), are known to cause respiratory and enteric symptoms. In the SARS outbreak of 2002-03, 16%-73% of patients with SARS had diarrhea during the course of the disease, usually within the first week of illness [5]. SARS-CoV ribonucleic acid (RNA) was only detected in stools from the fifth day of illness onwards, and the proportion of stool specimens positive for viral RNA progressively increased and peaked at day 11 of the illness, with viral RNA still present in the feces of a small proportion of patients even after 30 days of illness [6]. Till now, infection control and surveillance focus on the respiratory system. The ignorance of SARS-CoV-2 in the digestive system may cause difficulty with disease control. Gastrointestinal symptoms seem to be uncommon in patients with COVID-19 when compared with SARS [7-8]. However, they should not be ignored as the increasing rate of diarrhea occurs in confirmed COVID-19 patients according to a recent report that 14 of 138 confirmed patients had diarrhea [9]. Those early reports may not represent the actual rate of gastrointestinal symptoms caused by SARS-CoV-2 because in the early stages of the outbreak, the limited resources for detection were only provided to those patients with severe symptoms like respiratory distress syndrome. About 27% of SARS patients have diarrhea and since full-length genome sequences identified that SARS-CoV-2 is 79.5% identical to SARS-CoV and shares the same receptor angiotensin-converting enzyme 2 (ACE2), it is estimated that the rate of gastrointestinal symptoms would be higher in patients with COVID-19 [10]. One possible route for the movement of SARS-CoV-2 into the digestive system may be the “trachea-esophagus-ileum-colon” as single-cell transcriptome analysis showed ACE2, the entry receptor for SARS-CoV-2, highly expressed in lung AT2 cells, esophagus upper and stratified epithelial cells, and enterocytes from the ileum and colon [11].

## Review

Data and methods

Literature Search and Selection

We conducted a comprehensive systematic literature search of online databases, including PubMed, Embase, Web of Science, WanFang Data, and CNKI, from December 2019 to February 2020, to identify all case studies. The search terms and relative variants were as follows: COVID-19; 2019-nCoV; SARS-CoV-2; clinical characteristics; discharge rate; gastrointestinal infection; review. The inclusion criteria for the articles are as follows: study population: patients diagnosed with COVID-19; study design: case studies; measurement of results: at least one result that reported gastrointestinal symptoms and the presence of viruses in the stool.

Abstracts from conferences and commentary articles were excluded. Data extraction and quality assessment data extraction and the evaluation of literature quality were conducted independently by two investigators (R.E. and G.A.). Any disagreement was resolved by another investigator (C.M.)

Results

Natural History and Clinical Characteristics

Huang et al. first reported the clinical features of 41 patients confirmed to be infected with COVID-19 on January 2, 2020, which include 13 intensive care unit (ICU) cases and 28 non-ICU cases [7]. More than half of the cases (66%) had been exposed to the Huanan Seafood Wholesale Market. Almost all patients had bilateral lung ground-glass opacity on computed tomography imaging. The initial symptoms included fever (98%), cough (76%), dyspnea (55%), myalgia or fatigue (44%), sputum production (28%), headache (8%), hemoptysis (5%), and diarrhea (3%). Only one patient did not present fever in the early stage of the disease. Twelve (29%) cases progressed to acute respiratory distress syndrome (ARDS), five (12%) had acute cardiac injury, three (7%) had acute kidney injury (AKI), and three (7%) had shock. At the data cutoff date, 28 (68%) patients were discharged and six (15%) had died. On January 20, 2020, Chen et al. reported 99 cases with SARS-CoV-2-infected pneumonia [8]. This case series revealed that older males with comorbidities as a result of weaker immune function were the most susceptible to COVID-19 incidence. The symptoms, complications, and treatments in this study were similar to the previously published study by Huang and colleagues. At the data cutoff date, 31 (31%) of the patients were discharged, 11 (11%) died, and 57 (58%) were still hospitalized. A study by Li et al. reported on 425 COVID-19 cases in Wuhan confirmed between January 1 and 22, 2020 [12]. The mean incubation period was 5.2 days, with the 95th percentile of the distribution at 12.5 days, though uncertainty remains. Two subsequent studies confirmed the pattern of signs and symptoms [13-14]. At the time of this writing, the most recent published case series of 138 confirmed cases included 36 requiring intensive care by the data cutoff date of February 3, 2020 [9]. It also found the common presenting symptoms to be fever (136), fatigue (96), and dry cough (82), though there were two patients who did not present any signs of fever at the onset of illness. A higher proportion of cases presented with gastrointestinal symptoms, including diarrhea and nausea (14) initially presented one to two days prior to the development of fever and dyspnea. Forty-seven patients (34%) were discharged while six (4%) died, while the remainder were still hospitalized. Organ failure complications were similar to the original studies. Taken together, these studies indicate the main clinical manifestations of COVID-19 are fever (90% or more), cough (around 75%), and dyspnea (up to 50%). A small but significant subset had gastrointestinal symptoms.

Basic Virology

Coronaviruses are widespread in humans and several other vertebrates and cause respiratory, enteric, hepatic, and neurologic diseases. Notably, the severe acute respiratory syndrome coronavirus (SARS-CoV) in 2003 and Middle East respiratory syndrome coronavirus (MERS-CoV) in 2012 have caused human epidemics. A comparison with the current virus shows several significant differences and similarities. Both MERS-CoV and SARS-CoV have much higher case fatality rates (40% and 10%, respectively). Though the current SARS-CoV-2 shares 79% of its genome with SARS-CoV, it appears to be much more transmissible [15]. SARS-CoV-2 has been sequenced. A phylogenetic analysis found a bat origin for SARS-CoV-2. There is a diversity of possible intermediate hosts for SARS-CoV-2, including pangolins, but not mice and rats. There are many similarities of SARS-CoV-2 with the original SARS-CoV. Using computer modeling, researchers found that the spike proteins of SARS-CoV-2 and SARS-CoV have almost identical 3-D structures in the receptor-binding domain that maintains van der Waals forces. The SARS-CoV spike protein has a strong binding affinity to human ACE2 based on biochemical interaction studies and crystal structure analysis. SARS-CoV-2 and SARS-CoV spike proteins share 76.5% identity in amino acid sequences and, importantly, the SARS-CoV-2 and SARS-CoV spike proteins have a high degree of homology [16]. Both SARS-CoVs enter the cell via the ACE2 receptor [17]. The SARS-Cov-2 first predominantly infects lower airways and binds to ACE2 on alveolar epithelial cells. Both viruses are potent inducers of inflammatory cytokines. The “cytokine storm” or “cytokine cascade” is the postulated mechanism for organ damage. The virus activates immune cells and induces the secretion of inflammatory cytokines and chemokines into pulmonary vascular endothelial cells.

Transmission Dynamics and Protective Measures

The preliminary estimate of R0 (the expected number of cases directly produced by one person in a population susceptible to infection) for COVID-19 is 2.2 (95% CI, 1.4 to 3.9) [18]. Fomites are suspected as the main source of infectious particles, though some uncertainty remains. Other coronaviruses have been shown to persist for days on uncleaned surfaces [15]. Additionally, SARS-CoV-2 RNA was detected in the stool specimen in a person who had symptoms while the serum specimen tested negative [19]. Recently, SARS-Cov-2 was isolated from a swab sample of a confirmed patient’s feces by Chinese researchers, indicating the potential for fecal-oral transmission [20]. Studies have shown effective person-to-person transmission of 2019-nCoV even in the presence of isolation efforts in medical facilities [21-22]. The recent case series reported 57 (41%) of 138 patients were infected in hospital settings, including 40 (29%) medical staff [9]. Handwashing is the mainstay of viral control. Contact isolation gear, such as masks, gowns, and gloves, are also recommended. Transmission via the ocular surface is possible, so eye protection should also be used [23].

Enteric Involvement of Coronavirus

Novel coronavirus symptoms seem to be mostly focused on fever and cough, but gastrointestinal symptoms should be a new focus for clinicians, according to two new papers published online in Gastroenterology, Jinyang Gu et al. describe how investigators from Shanghai, China, sought to document the symptoms of the novel coronavirus. Although fever, dry cough, and dyspnea present in most cases, they wanted to understand what impact the virus had on symptoms such as diarrhea, nausea, vomiting, and abdominal discomfort. So far, those symptoms have varied among different study populations. Former studies on SARS, which is related to the coronavirus and can present with similar symptoms, showed that SARS was verified in patients after detection in biopsy specimens and stool. This was true even after the patients had been discharged from the hospital. The study authors noted that the first US patient admitted to a hospital with confirmed coronavirus had a loose bowel movement on hospital day two [19]. Labs in China have been able to isolate the live coronavirus from the stool of patients, the authors said (unpublished). These factors brought the gastrointestinal tract to the forefront of investigators’ minds and suggest that clinicians should identify patients with gastrointestinal symptoms and carefully monitor those patients [24].

Another similarity that may be noted between SARS and COVID-19 is that mild to moderate liver injury has existed in patients [25]. Xiao F et al. examined the viral RNA in feces from 71 patients with confirmed COVID-19 during their hospitalization from February 1-14, 2020 [26]. They collected serum, nasopharyngeal and oropharyngeal swabs, urine, stool, and tissues (from endoscopy) from the patients. The age of the patients ranged from 10 months to 78 years. The duration of positive stool tests ranged from 1 to 12 days, they added, and patients remained positive via stool tests after showing negative in respiratory samples. The researchers found that 53.4% of patients had SARS-CoV-2 RNA in their stool and 23% of patients tested positive in their stool despite testing negative for the virus in respiratory samples. This finding indicates that viral gastrointestinal infection and the potential fecal-oral transmission can last even after viral clearance in the respiratory tract. The author strongly recommends that rRT-PCR testing for SARS-CoV-2 from feces should be performed routinely in SARS-CoV-2 patients, and transmission-based precautions for hospitalized SARS-CoV-2 patients should continue if the feces tests positive by rRT-PCR testing. Table 1 provides a brief review of the study by Yu He et al. of COVID-19 while Table 2 summarizes all the included clinical studies to date [27-28].

**Table 1:** COVID-19: Coronavirus Disease 2019 Yu He MD et al. [27] SARS: Severe Acute Respiratory Syndrome

Items	COVID-19	COVID-19	COVID-19	SARS
Journal	Lancet	Lancet	JAMA	Lancet
Cases	41	99	138	1425
Published data	24-01-2020	2020/1/29	07-02-2020	24-03-2003
Fever (%)	98	83	98.6	94
Cough (%)	76	82	82	50.4
Shortness of breath (%)	55	31	31.2	30.6
Sputum production (%)	28	NA	26.8	27.8
Diarrhea (%)	3	2	10.1	27
Death (%)	15	11	4.3	<60 years old: 13.2%
				>60 years old: 43.3%

**Table 2:** Summary of included clinical studies to date CRRT: Continuous Renal Replacement Therapy; ARDS: Acute Respiratory Distress Syndrome; AKI: Acute Kidney Injury; NIV: Noninvasive Ventilation; IMV: Invasive Mechanical Ventilation; ECMO: Extracorporeal Membrane Oxygenation Jiang, F. et al. [28]

Author	Huang et al. [7]	Chen et al. [8]	Li et al. [12]	Song et al. [13]	Chen et al. [14]	Wang et al. [9]
Study setting	Wuhan Jinyintan	Wuhan Jinyintan	Hospitals in Wuhan	Shanghai Public Health Clinical Center	Tongji Hospital	Zhongnan Hospital of Wuhan University
	Hospital from Dec 16, 2019, to Jan 2, 2020	Hospital from Jan 1 to Jan 20, 2020	on Jan 22, 2020	from Jan 20 to Jan 27, 2020	from Jan 14 to Jan 29, 2020	from Jan 1 to Jan 28, 2020
						Follow-up to Feb 3, 2020
City	Wuhan, China	Wuhan, China	Wuhan, China	Shanghai, China	Wuhan, China	Wuhan, China
Total patients	41	99	425	51	29	138
Age, mean (IQR) or mean ± SD, years	49 (41–58)	55.5 ± 13.1	56 (26–82)	49 ± 16	56 (26–79)	56 (42–68)
Gender, male	30 (73%)	67 (68%)	31 (66%)	25 (49%)	21 (72%)	75 (54.3%)
Exposure history, cases	27 (66%) exposed to	49 (49%) exposed to	26 (55%) exposed to	50 (98%) exposed to	2 (7%) exposed to	12 (8.7%) exposed to
	Huanan Seafood	Huanan Seafood	Huanan Seafood	Wuhan	Huanan Seafood	Huanan Seafood
	Wholesale Market	Wholesale Market	Wholesale Market		Wholesale Market	Wholesale Market
X-ray and CT findings, cases	Bilateral ground-glass opacity, 40 (98%)	Multiple mottling and ground-glass opacity, 14 (14%)	Radiographic evidence of pneumonia	Ground glass opacity, 39 (77%)	NA	Ground-glass opacity, 138 (100%)
Signs and symptoms	Fever, 40 (98%)	Fever, 82 (83%)	Fever, with or without recorded temperature	Fever, 49 (96%)	Fever, 28 (97%)	Fever, 136 (98.6%)
	Cough, 31 (76%)	Cough, 81 (82%)		Cough, 24 (47%)	Cough or expectoration, (21 -72%)	Fatigue, 96 (69.6%)
	Myalgia or fatigue 18 (44%)	Shortness of breath, 31 (31%)		Phlegm, 10 (20%)	Dyspnea, 17 (59%)	Dry cough, 82 (59.4%)
	Sputum production, 11/39 (28%)	Muscle ache, 11 (11%)		Myalgia or fatigue, 16 (31%)	Myalgia or fatigue, 12 (41%)	Anorexia, 55 (39.9%)
	Headache, 3/38 (8%)	Confusion, 9 (9%)		Headache and dizziness, 8 (16%)	Headache, 2 (7%)	Myalgia, 48 (34.8%)
	Hemoptysis, 2/39 (5%)	Headache, 8 (8%)		Dyspnea or chest pain, 7 (14%)	Diarrhea, 4 (14%)	Dyspnea, 43 (31.2%)
	Diarrhea, 1/38 (3%)	Sore throat, 5 (5%)		Loss of appetite, 9 (18%)		Expectoration, 37 (26.8%)
	Dyspnea, 22/40 (55%)	Rhinorrhea, 4 (4%)		Diarrhea, 5 (10%)		Pharyngalgia, 24 (17.4%)
		Chest pain, 2 (2%)		Stuffy and runny nose, 2 (4%)		Diarrhea, 14 (10.1%)
		Diarrhea, 2 (2%)		Sore throat, 3 (6%)		Nausea, 14 (10.1%)
		Nausea and vomiting, 1 (1%)		Nausea and vomiting, 3 (6%)		Dizziness, 13 (9.4%)
						Headache, 9 (6.5%)
						Vomiting, 5 (3.6%)
						Abdominal pain, 3 (2.2%)
Complications	ARDS, 12 (29%)	ARDS, 17 (17%)	NA	NA	NA	Shock, 12 (8.7%)
	RNAemia, 6 (15%)	Acute renal injury, 3 (3%)				Acute cardiac injury 10 (7.2%)
	Acute cardiac injury, 5	Acute respiratory injury, 8 (8%)				Arrhythmia, 23 (16.7%)
	-12%	Septic shock, 4 (4%)				ARDS, 27 (19.6%)
	Acute kidney injury, 3 (7%)	Ventilator-associated pneumonia, 1 (1%)				AKI, 5 (3.6%)
Treatments	Antiviral, 38 (93%)	Oxygen therapy, 75 (76%)	NA	NA	NA	Antiviral, 124 (89.9%)
	Antibiotic, 41 (100%)	Noninvasive ventilation, 13 (13%)				Glucocorticoid, 62 (44.9%)
	Corticosteroid, 9 (22%)	Invasive ventilation, 4 (4%)				CRRT, 2 (1.45%)
	CRRT, 3 (7%)	CRRT, 9 (9%)				Oxygen inhalation
	Nasal cannula, 27 (66%)	ECMO, 3 (3%)				106 (76.81%)
	Noninvasive ventilation or high-flow nasal cannula, 10 (24%)	Antibiotic, 70 (71%)				NIV, 15 (10.9%)
	Invasive mechanical ventilation, 2 (5%)	Antifungal, 15 (15%)				IMV, 17 (12.32%)
		Antiviral, 75 (76%)				ECMO, 4 (2.9%)
		Glucocorticoids, 19 (19%)				

Discussion

This review included the latest studies from December 2019 to March 2019 to analyze the clinical characteristics of the novel coronavirus. New evidence supports the possibility of gastrointestinal infection with the SARS-CoV-2, as well as a possible fecal-oral route of transmission, according to the results of studies published in Gastroenterology. Currently, a SARS-CoV-2 infection causes primarily a respiratory-based constellation of symptoms. Due to the respiratory nature of symptoms, it has been hypothesized that the virus infects respiratory epithelial cells and is transmitted via respiratory droplets from human to human. However, viral target cells and organs have not been explicitly identified, creating a significant gap in the current understanding of the pathogenesis of SARS-CoV-2 and its transmission routes [24-26]. Moreover, a recent case report detected SARS-CoV-2 viral RNA in stool samples, thus challenging these hypotheses. Therefore, researchers examined stool samples obtained between February 1 and February 14, 2020, from 73 patients hospitalized as a result of SARS-CoV-2 infection for viral RNA. Results demonstrated that 53.42% of these patients had positive samples for SARS-CoV-2 RNA. The samples remained positive for a range of one to 12 days; of note, 23.29% of patients had positive stool samples after respiratory samples were negative for viral RNA. A study described one patient, a man, aged 78 years, who was admitted to a hospital in Guangdong Province, China, on January 17, 2020. rRT-PCR was positive for SARS-CoV-2, and the patient experienced 10 days of respiratory illness with the hallmarks of COVID-19. On day 10, however, he presented with coffee-ground gastric contents in his gastric drainage tube. Fecal occult blood testing indicated an upper gastrointestinal bleed; subsequent endoscopy found mucosal damage to the esophagus, and esophageal, gastric, duodenal, and colonic samples were taken for testing. Hematoxylin and eosin staining showed no significant damage, but numerous infiltrating plasma cells and lymphocytes, as well as interstitial edema, were seen in the lamina propria of the stomach, duodenum, and rectum. Most significantly, results demonstrated positive ACE2 protein staining, mainly in the cytoplasm of gastrointestinal epithelial cells. It has been previously shown that SARS-CoV-2 uses this protein as a viral receptor for its entry process. Immunofluorescence testing showed that ACE2 was abundantly expressed in the glandular cells of the gastric, duodenal and rectal epithelia of the above-mentioned patient, as well as other patients. These data demonstrated that infectious virions are being secreted from the gastrointestinal cells of people with SARS-CoV-2 infection, and thus support the potential for fecal-oral transmission of the virus. The discharge guideline depending on the respiratory tract test also meets the challenge.

Interestingly, while the virus test of the nasopharyngeal swab switched from positive to negative after the treatment, the rectal swab specimens still tested positive [29]. These cases remind the clinicians that the rectal swab may be equally important to the pharyngeal swab even if the patient is asymptomatic, which challenges the latest published guideline provided by National Health Commission of China that two successive, negative respiratory tract tests are regarded as the standard for discharge and termination of compulsory isolation for COVID-19 patients [30]. Famous well-described clusters of infection of SARS in Amoy Gardens, Hong Kong, drew the attention of health officials on fomite transmission because two-thirds of the confirmed SARS patients in Amoy Gardens had diarrhea [31]. As findings showed that patients with SARS could discharge SARS-CoV in their stool up to 73 days after symptom onset, the stools with the virus became the resource of contamination of airdrops and a variety of environmental surfaces, which may contribute to the clusters of infection [32]. Similarly, evidence showed that SARS-CoV-2 were identified in four stool specimens (four out of 62), so fomite transmission should not be ignored in the transmission of SARS-CoV-2 since the virus may move from the respiratory tract into the gastrointestinal tract the recovered patients may discharge the stool with the virus for a long time [33]. According to a recently published report by the Centers for Disease Control and Prevention (CDC) of China, community-acquired infections are becoming the predominant route in transmission [34]. Based on these cases and the lessons from SARS, rRT-PCR testing for SARS-CoV-2 from feces should be performed in SARS-CoV-2 patients before discharge.

## Conclusions

Clinicians should recognize that digestive symptoms, such as diarrhea, may be a presenting feature of COVID-19, and that the index of suspicion may need to be raised earlier in at-risk patients presenting with digestive symptoms rather than waiting for respiratory symptoms to emerge. It should also be considered before discharge that viral gastrointestinal infection and potential fecal-oral transmission can last even after viral clearance from the respiratory tract.

## Appendices

What this study adds

- A significant portion of coronavirus patients experiences diarrhea, nausea, vomiting, and/or abdominal discomfort before the onset of respiratory symptoms.

- Viral RNA is detectable in fecal samples from suspected cases, indicating that the virus sheds into the stool.

- Viral gastrointestinal infection and potential fecal-oral transmission can last even after viral clearance from the respiratory tract.
